# Missed opportunities for delivering nutrition interventions in first 1000 days of life in India: insights from the National Family Health Survey, 2006 and 2016

**DOI:** 10.1136/bmjgh-2020-003717

**Published:** 2021-02-24

**Authors:** Phuong Hong Nguyen, Rasmi Avula, Lan Mai Tran, Vani Sethi, Alok Kumar, Dinesh Baswal, Nemat Hajeebhoy, Alok Ranjan, Purnima Menon

**Affiliations:** 1 Poverty, Health and Nutrition Division, International Food Policy Research Institute, Washington, District of Columbia, USA; 2 FHI Solutions, Durham, North Carolina, USA; 3 UNICEF, Delhi, India; 4 Department of Health & Family Welfare, Government of Uttar Pradesh, Formerly with NITI Aayog, New Delhi, Delhi, India; 5 Formerly with the Maternal Health Division, India Ministry of Health and Family Welfare, New Delhi, Delhi, India; 6 Bill & Melinda Gates Foundation, Seattle, Washington, USA; 7 Bill & Melinda Gates Foundation, Delhi, India

**Keywords:** nutrition, health systems, health services research, public health, cross-sectional survey

## Abstract

**Objectives:**

Existing health and community nutrition systems have the potential to deliver many nutrition interventions. However, the coverage of nutrition interventions across the delivery platforms of these systems has not been uniform. We (1) examined the opportunity gaps between delivery platforms and corresponding nutrition interventions through the continuum of care in India between 2006 and 2016 and and (2) assessed inequalities in these opportunity gaps.

**Methods:**

We used two rounds of the National Family Health Survey data from 2005 to 2006 and 2015–2016 (n=36 850 and 190 898 mother–child dyads, respectively). We examine the opportunity gaps over time for seven nutrition interventions and their associated delivery platforms at national and state levels. We assessed equality and changes in equality between 2006 and 2016 for opportunity gaps by education, residence, socioeconomic status (SES), public and private platforms.

**Results:**

Coverage of nutrition interventions was consistently lower than the reach of their associated delivery platforms; opportunity gaps ranging from 9 to 32 percentage points (pp) during the pregnancy, 17 pp during delivery and 9–26 pp during childhood in 2006. Between 2006 and 2016, coverage improved for most indicators, but coverage increases for nutrition interventions was lower than for associated delivery platforms. The opportunity gaps were larger among women with higher education (22–57 pp in 2016), higher SES status and living in urban areas (23–57 pp), despite higher coverage of most interventions and the delivery platforms among these groups. Opportunity gaps vary tremendously by state with the highest gaps observed in Tripura, Andaman and Nicobar islands, and Punjab for different indicators.

**Conclusions:**

India’s progress in coverage of health and nutrition interventions in the last decade is promising, but both opportunity and equality gaps remained. It is critical to close these gaps by addressing policy and programmatic delivery systems bottlenecks to achieve universal coverage for both health and nutrition within the delivery system.

**Key questions**

**What is already known?**

- Existing health and community-based platforms have the potential to deliver nutrition interventions along the continuum of care during the first 1000 days of life.
- In India, the Integrated Child Development Services and the National Health Mission are the two flagship programmes that deliver these interventions. Some interventions act as gateways/platforms for delivering other nutrition interventions. However, the coverage of nutrition interventions remains suboptimal.

**What are the new findings?**

- Between 2006 and 2016, coverage of community-based platforms improved significantly, but only reached approximately half of the population, thus still far from achieving the goal of universal coverage.
- Coverage of nutrition interventions was consistently lower than the reach of their associated delivery platforms. The opportunity gaps are large and vary tremendously by women’s age, educational status, place of residence, wealth status and by geographical location.

**What do the new findings imply?**

- Our findings provide insight into potential priorities for improvement in different segments of the population. There is a need to improve the integration of nutrition interventions to close opportunity gaps for the well-off groups. At the same time, improving the reach of both platforms and nutrition interventions is essential for disadvantaged groups.
- Both opportunity and equality gaps must be closed by assessing and tracking policy, fiscal and programmatic health systems bottlenecks to achieve universal coverage for both health and nutrition.

## Introduction

Nutrition is central to the Sustainable Development Goals (SDGs) agenda, and at least 12 of the 17 SDGs include indicators relevant for nutrition. Investing in actions to accelerate the reduction of all forms of malnutrition is therefore critical to support the achievement of global targets. Such accelerations are only possible with universal coverage of effective nutrition interventions to all population groups,^1^ particularly in low-income and middle-income countries where mortality and undernutrition are concentrated.^2^

Nutrition interventions can be delivered through several platforms such as health, agriculture, social protection and market-based programmes.^3^ Among these, health systems are the most effective platform to reach women and children in the first 1000 days and along the continuum of care.^4^ Within health systems, nutrition interventions can be delivered during antenatal care (ANC) (eg, micronutrient and food supplementation, deworming, nutrition counselling), during intrapartum care (eg, delayed cord clamping and support for early initiation of breastfeeding (EIBF)) and during postnatal care and childhood (eg, counselling for breast feeding and complementary feeding, micronutrient and food supplementation, and management of child illness).^5 6^ Missing the opportunity to deliver nutrition interventions through its associated health platforms has been defined as ‘opportunity gaps’^4^ and examining these gaps can help to identify opportunities to influence health systems for nutrition more effectively. A recent study from 81 low-income and middle-income countries suggested that improvement in quality of care among health systems to effectively deliver a core subset of 19 maternal and child interventions could reduce maternal and neonatal mortality by 28% and reduce stillbirth by 22%.^7^ Despite a strong evidence of effective delivery platforms and high coverage impacts, universal coverage for most essential interventions is still far from optimal with slow progress, poor quality and high inequalities within and between countries.^7^

In India, a highly populated country, which contributes to a third of the global burden of undernutrition, several evidence-based nutrition interventions in the first 1000 days are delivered through two major national programmes: (1) the Integrated Child Development Services (ICDS) scheme managed by the Ministry of Women and Child Development and (2) the National Health Mission (NHM) managed by the Ministry of Health and Family Welfare. While food supplementation and growth monitoring interventions are delivered through the ICDS programme, micronutrient supplementation, deworming, and curative interventions are delivered through the NHM, and behaviour change communication is delivered through both the programmes.^8 9^ These two programmes are implemented across all the states with a universal reach mandate and therefore the interventions have the potential to attain 100% coverage. However, there is a gap in the coverage of interventions and limited evidence exists on whether nutrition interventions are effectively delivered through these existing community-based platforms. Our study aims to (1) examine the opportunity gaps between the reach of the delivery platforms and the coverage of nutrition interventions through the continuum of care (from pregnancy up to early childhood) and (2) assess the levels of inequalities in these opportunity gaps.

## Methods

### Data sources

We used nationally representative data from the third round of the National Family Health Survey in 2005–2006 (NFHS-3)^10^ and the fourth round in 2015–2016 (NFHS-4).^11^ These cross-sectional surveys follow a systematic, multistage stratified sampling design, covering all states/union territories in India. NFHS-3 surveyed 109 041 households and was representative at the state level; NFHS-4 surveyed 601 509 households, representative at both district and state level. The response rate was high for both survey rounds (94.5% in NFHS-3% and 96.7% in NFHS-4 for the primary female respondents). We analysed data from mother–child dyads with the last child aged 0–5 years (n=36 850 for NFHS-3 and 190 898 for NFHS-4).

### Indicators

We used available data for a set of seven nutrition interventions during the pregnancy, delivery, postnatal and early childhood and to identify the platforms for their delivery. During the pregnancy, ANC is considered the critical platform to deliver iron and folic acid (IFA) supplementation and deworming. Food supplementation is the gateway platform to deliver nutrition and health counselling during both pregnancy and lactation. At the time of birth, institutional delivery provides an opportunity for health staff to provide counselling and support for EIBF. As data on breastfeeding counselling/support during the time of delivery is unavailable, we used EIBF as a proxy for it, although it is plausible that coverage of counselling could be higher than the actual behaviour. We applied the standard WHO definition^12^ to construct the EIBF indicator (proportion of infants who were put to the breast within 1 hour of birth) for both rounds of survey data. During early childhood, routine growth monitoring is a potential platform to deliver nutrition counselling on child growth. For children suffering from diarrhoea, oral rehydration salts (ORS) could be a platform to deliver zinc as well (children should be provided with 20 mg per day of zinc supplementation for 10–14 days or 10 mg per day for infants under the age of 6 months), which is considered efficacious in treating diarrhoea.^13^

Detailed indicator definitions for delivery platforms and nutrition interventions are presented in table 1. We consider the difference between the reach of a platform and the coverage of a nutrition intervention through that platform to be the opportunity gap for attaining the maximum possible coverage for that intervention.

**Table 1:** Indicators for delivery platforms, nutrition interventions and opportunity gap

Delivery platform	Nutrition intervention	Opportunity gap
**During pregnancy**		
Early ANC Percentage of mothers who received ANC during the first trimester of pregnancy for the last birth in the last 5 years.	Consumption of IFA supplements Percentage of mothers who took IFA supplements for at least 100 days for the last birth in the last 5 years.	Early ANC: IFA 100+ Percentage of mothers who received early ANC but did not consume 100 IFA tablets during pregnancy.
At least 4 ANC visits Percentage of mothers who received at least four ANCs for the last birth in the last 5 years.	Deworming during pregnancy Percentage of mothers who received deworming for the last birth in the last 5 years.	ANC 4+: deworming Percentage of mothers who received at least four ANCs but did not receive deworming during pregnancy.
Food supplementation during pregnancy Percentage of mothers with children under age 5 years who received THR from the AWC during pregnancy.	Health and nutrition education during pregnancy Percentage of mothers with children under age 5 years who received health and nutrition education during pregnancy from the AWC.	THR: nutrition counselling Percentage of mothers who received THR but did not receive health and nutrition education during pregnancy.
**During delivery**		
Institutional delivery Percentage of mothers with children under age 5 years who delivered in a health facility.	EIBF: Percentage of infants 0–24 months who were put to the breast within 1 hour of birth.	Institutional delivery: EIBF Percentage of mothers who delivered in a health facility but did not practice EIBF.
**During lactation**		
Food supplementation during lactation Percentage of mothers with children under age 5 years who received THR from the AWC during lactation.	Health and nutrition education during lactation Percentage of mothers with children under age 5 years in areas who received health and nutrition education during lactation from the AWC.	THR: Nutrition counselling Percentage of mothers who received THR but did not receive health and nutrition education during lactation.
**During childhood**		
Weight monitoring Percentage of children less than 5 years who were weighed.	Weight counselling after weighing Percentage of mothers with children less than 5 years who received counselling after their children being weighed.	Weight: weight counselling Percentage of mothers whose children less than 5 years were weighed but did not received weight counselling.
ORS during diarrhoea Percentage of children less than 5 years with diarrhoea in the last 2 weeks who received ORS.	Zinc during diarrhoea Percentage of children less than 5 years with diarrhoea in the last 2 weeks who received zinc.	ORS: Zinc Percentage of children less than 5 years with diarrhoea in the last 2 weeks who received ORS but did not received zinc.

ANC, antenatal care; EIBF, early initiation of breast feeding; IFA, iron and folic acid; ORS, oral rehydration salts.

### Statistical analyses

We calculated the coverage and opportunity gaps at the national and state levels. We then conducted regression analyses to examine the changes over time between 2006 and 2016 in coverage and opportunity gaps, adjusted for the cluster sampling design and sampling weights used in the survey. We then examined equality and changes in equality in opportunity gaps by age groups (adolescence: 15–19 years, young adulthood: 20–24 years and adulthood: 25–49 years), education (no education vs primary education and at least high school) and intersection between socioeconomic status (SES) (lowest quintile, Q1 vs highest quintile, Q5) and residence (urban/rural). Household SES index was constructed using principal component analyses including housing structure, access to services (electricity, gas, water and sanitation services) and household assets.^14 15^ The first component derived from component scores was used to divide household SES into quintile. We used a data depiction called equiplot to visualise the coverage of health platform and nutrition interventions, as well as the opportunity gaps (indicating by the spread of the dots). We then used the slope index of inequality (SII) to examine wealth and education inequalities for each coverage and opportunity gaps.^16 17^ All analyses were performed using Stata V.15.1 and were adjusted for the cluster sampling design and sampling weights used in the survey.

### Patient and public involvement statement

As our study used a national-level secondary dataset, the women and their children who participated in the data collection were not involved in the design of the study and in the development of the research question and outcome measures.

## Results

### Opportunity gaps by the life stage in the continuum of care

**Figure 1:**
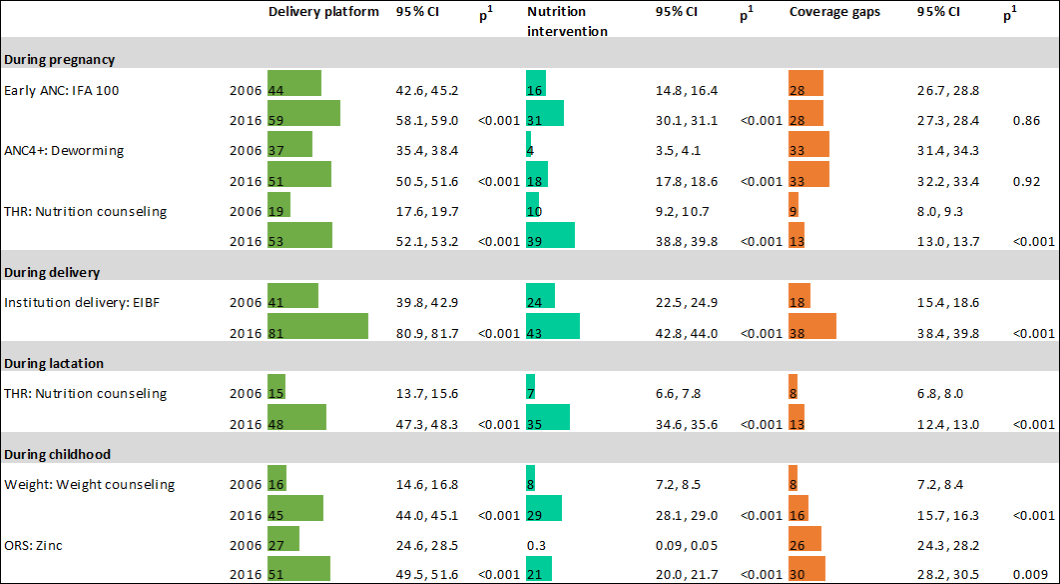
Estimates of coverage of nutrition interventions and their respective service delivery platforms in India, 2006 and 2016. ANC, antenatal care; EIBF, early initiation of breastfeeding; IFA, iron and folic acid; ORS, oral rehydration salts.

Coverage of nutrition interventions was consistently lower than the reach of the delivery platforms (figure 1) in both 2006 and 2016. For example, the coverage of IFA consumption (100+ tablets) was about a third of early ANC reach in 2006 (16 vs 44%) and about half in 2016 (31 vs 59%). The coverage of deworming was 4 vs 37% of 4 ANC in 2006, and 18 vs 51% in 2016. In 2006, the absolute opportunity gaps ranged between 9 and 33 percentage points (pp) during pregnancy, 18 pp during delivery and 8–26 pp during childhood. While food supplementation during pregnancy and lactation only reached <20% of women, coverage of nutrition counselling was only at 7%–10%. Weighing children and counselling after weighing were also low (16% and 8%, respectively). Among children who had diarrhoea in the previous 2 weeks, 27% had ORS but very few (0.3%) had zinc.

Between 2006 and 2016, the coverage for nutrition interventions and the delivery platforms related to these interventions improved significantly (all p<0.001); however, only half the population was reached. The only exception was institutional delivery that reached 81%. Despite improvements in reach, the opportunity gaps remained similar or even became wider, especially between institutional delivery and EIBF (from 18 pp gap in 2006 to 38 pp gap in 2016) (figure 1).

### Opportunity gaps by different age groups

**Figure 2:**
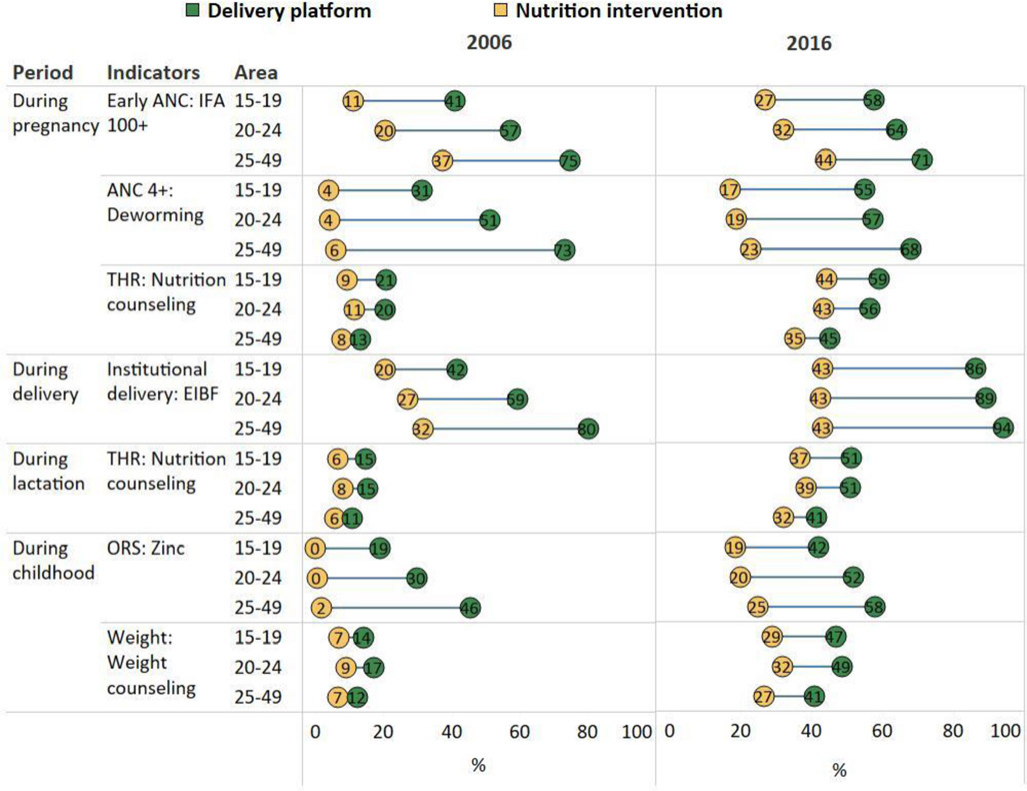
Opportunity gaps for different age groups in India, 2006 and 2016. ANC, antenatal care; EIBF, early initiation of breast feeding; IFA, iron and folic acid; ORS, oral rehydration salts.

In 2006, adolescent mothers had poorer access to ANC services and were less likely to deliver in health facilities compared with adult mothers (figure 2). They also had lower coverage of IFA consumption and EIBF. The opportunity gaps were narrower among adolescents compared with adult women: 20 vs 36pp for ANC and IFA consumption, 26 vs 67 pp for ANC and deworming, and 22 vs 48 pp for institutional delivery and EIBF. The reach of food supplementation, weighing children and nutrition counselling were similarly low among all age groups; the opportunity gaps between the coverage of nutrition counselling and the reach of its associated delivery platform were small for all age groups (5–12 pp).

**Figure 3:**
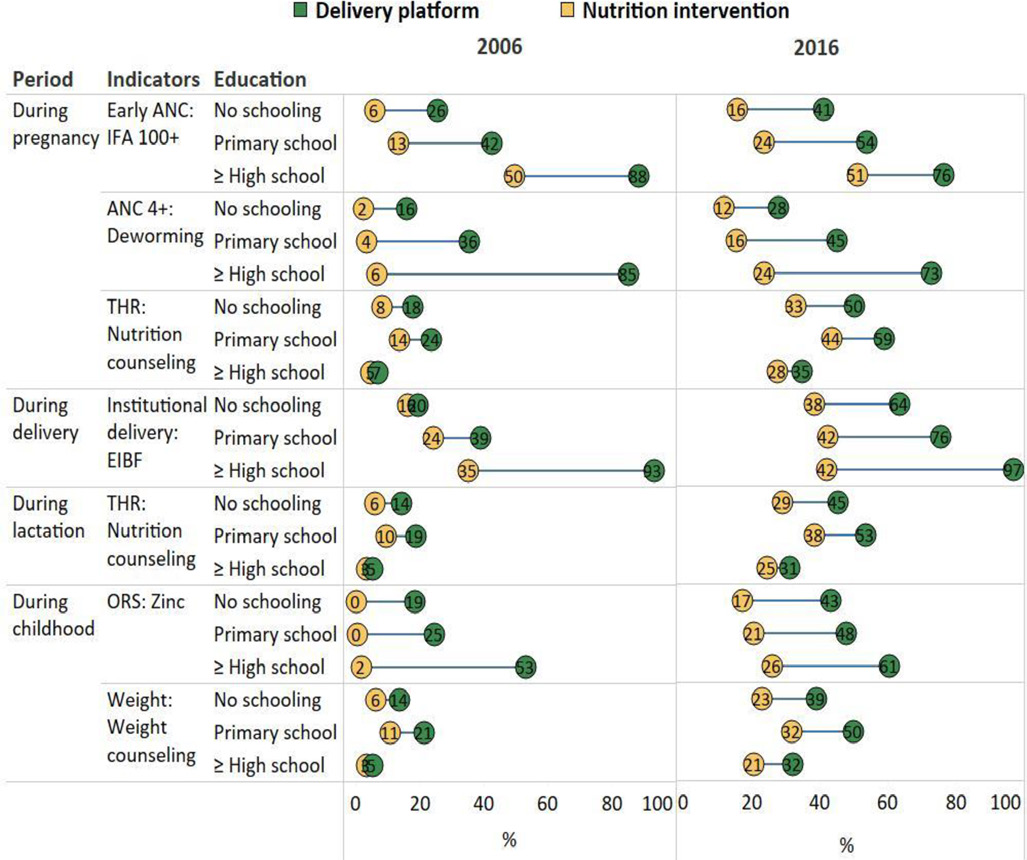
Opportunity gaps for different levels of education in India, 2006 and 2016. ANC, antenatal care; EIBF, early initiation of breast feeding; IFA, iron and folic acid; ORS, oral rehydration salts.

Between 2006 and 2016, coverage improved for most indicators (except for early ANC and four ANC visits among adult women), but coverage is still lower for adolescent groups (except for EIBF which are similar for all three age groups). The opportunity gaps became narrower for ANC and IFA consumption or deworming, but wider for institutional delivery and EIBF.

Opportunity gaps were higher among wealthier compared with poorer households across all age groups, as shown by the positive SII (in 2016, SII ranged from 5 to 29 pp for gap between early ANC and IFA 100+, 54 to 64 pp for gap between ANC 4+ and deworming, 30–39 pp for gap between institutional delivery and EIBF, and 26–46 pp for gap between ORS and zinc) (online supplemental table 1). In contrast, SIIs were negative for gaps between food supplementation and nutrition counselling, indicating the higher coverage gaps among the poorer population. The inequality decreased between 2006 and 2016 for most indicators in all age group, but unchanged for Take-home rations (THR) and nutrition counselling.

10.1136/bmjgh-2020-003717.supp1 Supplementary data

### Opportunity gaps by different levels of education

In 2006, women with no schooling had very low reach of delivery platforms (all <20%) and even lower coverage of nutrition intervention (0%–18%). When comparing the group of women with no schooling to those with at least high school education, we observed higher coverage but wider opportunity gaps among higher education group (figure 3 and online supplemental table 2). For example, in 2006, ANC coverage was 85% in high education vs only 16% in low education groups, institutional delivery coverage was 93% vs 20%, respectively. The opportunity gaps between early ANC and IFA consumption was 38 pp in high education vs 19 pp in low education groups, between ANC and deworming was 79 pp vs 14 pp, respectively, and between institutional delivery and EIBF was 58 pp vs 4 pp, respectively. The corresponding SIIs for these indicators were positive and ranged from 32 to 68 pp.

Between 2006 and 2016, coverage improved for most indicators in both low and high education groups. However, among women with no education, the reach of most delivery platforms and coverage of nutrition interventions were still low (all <50%, except for institutional delivery at 64%). Compared with 2006, the opportunity gaps became narrower in high education group but were wider in the low education group in 2016. The reach of ANC declined among higher education women (from 85% to 73%).

In contrast with other indicators, the opportunity gaps between food supplementation, weighing of children and nutrition counselling were larger among low education group. This is due to higher reach of the platform but similar coverage of nutrition intervention among low education, compared with higher education groups. Among high education group, the reach of the platform and coverage of interventions were both low and therefore the gap was small.

### Opportunity gaps by different levels of SES and residence

**Figure 4:**
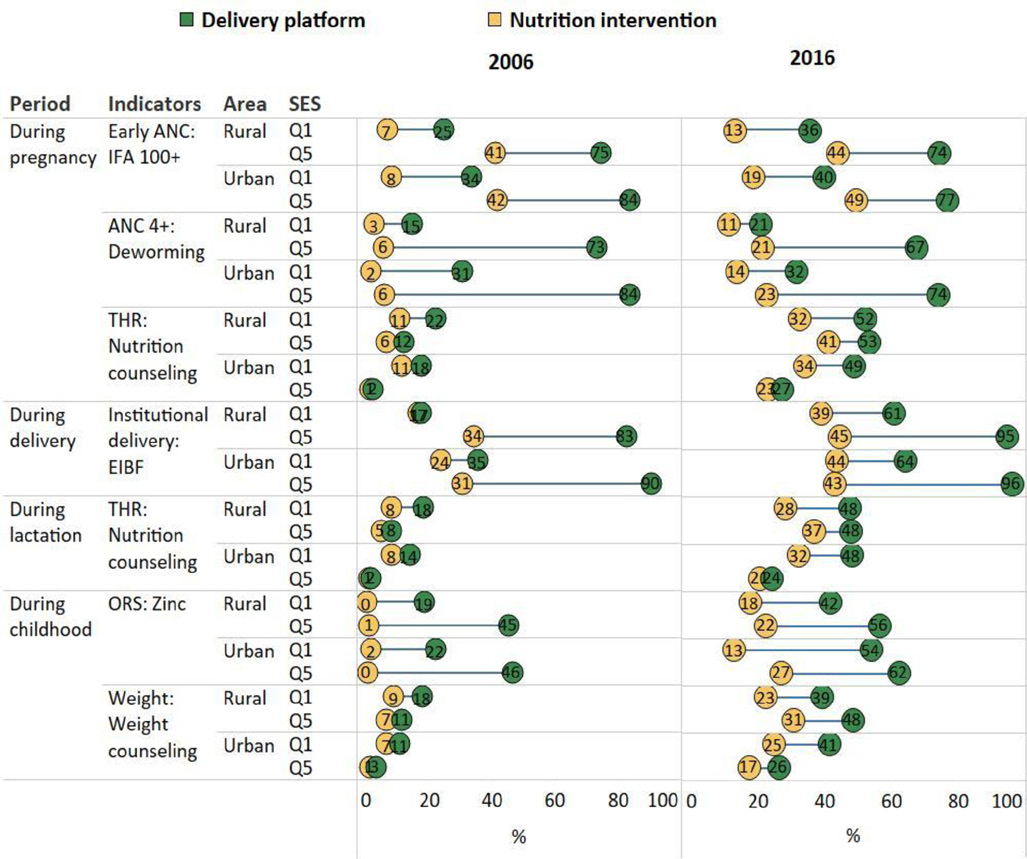
Opportunity gaps by residence and wealth quintile in India, 2006 and 2016. ANC, antenatal care; EIBF, early initiation of breast feeding; IFA, iron and folic acid; ORS, oral rehydration salts.

In 2006, the reach of the delivery platforms (except for food supplementation and weight monitoring among children) was lowest among the poor (quintile-Q1) living in rural areas, followed by the poor living in urban areas, and much higher among the rich (Q5) in both areas (figure 4 and online supplemental table 3). The coverage of nutrition interventions was also lower among the poor than the rich, but the difference was smaller for place of residence. The opportunity gaps were largest for the urban rich (42 pp between early ANC and IFA consumption, 78 pp between ANC and deworming, 60 pp between institutional delivery and EIBF), followed by rural rich (34 pp, 67 pp and 49 pp, respectively), and much smaller for the poor (8–29 pp).

Between 2006 and 2016, coverage improved for most delivery platforms and nutrition interventions for different socioeconomic groups in both rural and urban areas, except ANC which has been reduced among the rich (from 73% to 67% in rural areas and 84% to 74% in urban areas). Births in health facilities increased significantly, reaching 77% and 90%, respectively, in 2016, but EIBF increased at much slower rate, reaching only 39%–45%. Compared to 2006, most of the opportunity gaps in 2016 were narrower among different segment of the society, with wealth gaps reducing in all residential areas (SII ranged 16–52 pp in 2006 and 4–41 pp in 2016).

The pattern of food supplementation, weighing children and nutrition counselling was different from other indicators where the coverage was higher, and the gaps were larger among the rural poor compared with the urban rich.

### Opportunity gaps by states

Using data in 2016, we also examine the opportunity gaps for different states. In most of the states, high ANC coverage did not translate into high IFA or deworming coverage (online supplemental figure 1). For instance, in Kerala, despite 90% coverage of ANC4+ and 75% of women reporting consuming 100+ IFA tablets, only 22% received deworming. The states with lowest reach of ANC were Bihar and Nagaland (<15%) where the coverage of nutrition interventions was also low.

The opportunity gaps between institutional delivery and EIBF also varied widely by states (online supplemental figure 2a). Goa and Sikkim are the two states with higher coverage of institutional delivery (>95%) and also had high coverage of EIBF (73%–77%). Other states (such as Punjab and Andhra Pradesh) also had nearly universal coverage of institutional birth (>90%), but only had <40% EIBF coverage. Nagaland is the states with the lowest coverage of institutional birth at 36% but had relatively higher EIBF at 55%.

Regarding food supplementation and nutrition counselling, Odisha and Chhattisgarh stood out as two states with low opportunity gaps because they have high reach of delivery platform (84%–85%) and coverage of nutrition interventions (71%–76%) (online supplemental figure 2b). The poor performing states were Nagaland and Delhi where both delivery platform and interventions reached <10% of women. Similar findings were observed for the gaps between weighing children and counselling after weighing (online supplemental figure 3a).

Among children who had diarrhoea in the last 2 weeks, Goa and Meghalaya had lowest opportunity gaps between delivery platform (ORS) and nutrition intervention (zinc), with coverage reaching >50% for both. Sikkim had moderate high ORS coverage (64%), but lowest coverage of zinc (2%) (online supplemental figure 3b)

## Discussion

India’s community-based platforms, cutting across the continuum of care from maternal to child health during the first 1000 days of life, provide important opportunities to integrate and deliver a wide range of nutrition interventions. Although the reach of these platforms and coverage of nutrition interventions improved significantly between 2006 and 2016, the goal of universal coverage is still far from achieved. Most of these platforms only reached approximately half of the population with substantial inequality among the poor, those living in rural areas or having low education, and adolescent mothers. The coverage of nutrition interventions delivered through those platforms is even lower. The opportunity gaps between interventions and their associated platforms are large and vary tremendously by women’s age group, educational status, place of residence, wealth status and by geographical areas.

Delivery of many nutrition interventions depends on a strong health system with the assumption that the platforms has high coverage and good quality. In India, the ICDS and NHM are the two main programmes with some interventions acting as gateways/platforms for delivering other nutrition interventions. Although these platforms are well targeted to women and children, their potential to effectively deliver nutrition interventions is largely dependent on the functionality of the platform, the reliability and consistency of supplies, inputs and well-trained health staff, and the regular use of the services by beneficiaries.^3^ The reach for most platforms in 2016 was only at 45%–50% (except for institutional delivery at 81%), potentially due to both supply-side challenges (such as lack of human resources and/or training, gaps in provisioning of basic physical infrastructure and supply change^18^ and demand-side barriers (such as inequality toward marginalised sections of the population.^19–21^ Studies have also shown that not all nutrition interventions receive fiscal prioritisation in health annual budget plan allocations, with several information gaps in tracking disbursements and expenditure.^22^ Attention to convergent action at the national level focusing on a range of systems strengthening efforts, together with subnational implementation processes and special care for all marginalised sections of the population are critical to improve coverage, consistency, intensity and quality of interventions.

We found that the coverage of nutrition interventions falls far below the reach of its associated platform. For examples, the gaps between use of ANC and IFA consumption and deworming were at 21 pp and 33 pp, respectively, and unchanged overtime. This could be because of lack of IFA supplies or deworming medicine, inadequate focus on reviewing the intervention coverage, lack of behavioural change communication on use of IFA, or some other demand-side constraints. The important gaps in IFA supply have been documented including stock-outs, lack of personnel, procurement, storage and unsystematic distribution.^23^ Prior research has also shown that IFA consumption was strongly associated with counselling during ANC visits, maternal knowledge, beliefs, self-efficacy, positive social norms and support from family members.^24^ Our findings align well with existing literature that highlights the role of education and wealth for compliance to IFA recommendations.^24–26^

Our findings are aligned with evidence from analyses of 50 countries showing that global movements to scale up effective nutrition interventions and achieve universal health coverage have not been connected to reach their full potential.^4^ Overall review of 36 studies also showed large gaps between contact coverage and quality-adjusted coverage levels (10–38 pp) across the continuum of care for reproductive, maternal, newborn and child health.^27^ Therefore, closing the opportunity gap by increasing nutrition intervention coverage among those already reached by other platforms should be an immediate priority. A recent study from Malawi estimated that delivering nutrition interventions (such as iron interventions, nutrition counselling and breastfeeding counselling) consistently within the existing level of coverage would decrease prevalence of low birth weight by 3 pp (from 14% to 11%) and increase in EIBF by 10 pp (from 76% to 86.0%).^28^ In our study, the relative improvements between the reach of the platforms and the coverage of nutrition interventions were similar, which reiterates the potential of the platforms to achieve universal coverage of the interventions.

From an equity lens, health and nutrition services across the continuum of care are more widely used among women with higher education, better economic status and living in urban areas but the opportunity gaps were also larger among these subgroups. This reflects that the efforts of the programmes to expand and create incentives to use the platforms were more likely to reach the more advantaged group, but the reach of nutrition interventions was of concern for all groups. It also likely reflects the fact that several nutrition interventions, including those integrated in design into the health system, have been prioritised for delivery in rural areas. This could have been because early expansion of the National Rural Health Mission (now the NHM) created more opportunities in rural areas than in urban areas. Similarly, the ICDS platform is more wide-reaching in rural than in in urban areas, again, likely due to expansions in rural areas in the years between 2006 and 2016.^19^

Our findings provide insight into potential priorities for improvement among different segments of the population, such as improving nutrition interventions to close the opportunity gaps for the well-off group but improving the reach of both platform and nutrition interventions for the disadvantaged group. The gap between the reach of platforms and coverage of interventions also varies by types of intervention. Because of self-targeting programme platforms like the ICDS,^19 29^ women in the poorest quintile or had lower education were better reached by food supplementation, however, the opportunity gaps widened over the decade studied, likely reflecting quantitative expansions in the reach of basic programme platforms, but less functional integration of the full range of interventions.^30^

We also found large variability in opportunity gaps by states and districts for different outcomes. While some states have successfully closed opportunity gaps, achieving high platform reach and nutrition coverage, others still lag. For example, Lakshadweep closed the opportunity gap for IFA during ANC (both of them are >80%), Bihar and Nagaland had low coverage for both platforms (<15%), and Tripura, West Bengal and Jammu and Kashmir had opportunity gaps of ~50 pp. This could be driven by larger state-level implementation challenges or could be more local and concentrated in certain geographies within a state. Lessons should be shared from the successes of high-performing states on scaling up health services and integrating nutrition interventions within them. Focused strategic planning and action is needed to minimise regional disparities. India’s Aspirational Districts Programme^31^ provides additional workforce, partnerships, monitoring and fiscal envelope for the most disadvantaged districts, which could also be a tremendous opportunity to reduce opportunity gaps once platform are delivering.

Today, India has a robust policy landscape for maternal and child health and nutrition in the first 1000 days. The ANC platform has gained renewed attention under the Pradhan Mantri Surakshit Matritva Abhiyan,^32^ a fixed day monthly event to provide assured, comprehensive and quality ANC, free of cost, to all pregnant women. The Anaemia Mukt Bharat programme has set targets for India to reduce anaemia among pregnant women from 50% in 2016 to 35% in 2022 through providing anaemia prevention services including supplying IFA through routine and special ANC contacts.^33^ POSHAN Abhiyaan,^34^ the National Nutrition Mission, aims to address malnutrition in a mission-mode through strengthening implementation of the core health and nutrition interventions along with improving several social determinants. Behavioural change communication and technology are two of the core pillars of the mission and are being used together to deliver key nutrition messages. This provides an opportunity to increase the coverage of counselling interventions. In addition to this enabling environment, addressing the opportunity gap between delivery platforms and nutrition interventions would require simultaneous work at different levels, including a capable bureaucracy, both technical and administrative leadership, adequacy and flexibility of financing models and improving the use of data, and the expansion of state-level innovations to address state-specific challenges and gaps.^35 36^

Our study has some unique strengths. Using two large recent nationally representative surveys containing rich data on health and nutrition outcomes, we have applied innovative analytic approach to track and assess opportunity gaps in scaling up nutrition through health systems in India over period of 2006–2016 at the national and state levels. By examining the coverage of both platforms and nutrition interventions for multiple populations (adolescents vs adults, varying levels of education and intersection between residence and wealth quintile), we provide a potential tool to assess the potential for the health system by highlighting underserved populations. We acknowledge the limitation that our empirical analyses do not provide insights into how the changes in coverage of platform and nutrition occurred but this would require an expansive state-by-state or intervention-by-intervention analysis, which is beyond the scope of this paper. Our measurement of coverage used data for last-born children born in the 5 years preceding the survey. This could be prone to maternal recall bias given the long period of recall for the older children in the sample. We conducted sensitivity analyses to compare findings based on the sample of mothers with their last birth in the 1-year preceding the survey and observed similar findings; therefore, we retained the larger sample size available for births in the 5 years preceding the survey. Data gaps are another limitation: Of the 15 major nutrition interventions in India’s policies and programmes,^37^ nationally representative data is only available for seven of them, thus limiting our ability to analyse gaps for all interventions. Investing in data systems can help to close gaps in the data available to analyse coverage and opportunity gaps.^4^

## Conclusion

India’s progress in coverage of health and nutrition interventions in the last decade is promising, but both opportunity and equality gaps must be closed by assessing and tracking policy, fiscal and programmatic health systems bottlenecks to achieve universal coverage for both health and nutrition. The community-based platforms of India’s health and ICDS programmes offer tremendous potential and opportunity but seizing these opportunities will require careful investments in strategy and implementation.
