# Supplementary material for: Missed opportunities for delivering nutrition interventions in first 1000 days of life in India: insights from the National Family Health Survey, 2006 and 2016
Source: BMJ Glob Health. 2021 Feb 24;6(2):e003717. doi: 10.1136/bmjgh-2020-003717 (PMC7908280; doi:10.1136/bmjgh-2020-003717)
Supplement: Supplementary data [file bmjgh-2020-003717supp001.pdf]

Supplemental Table 1: Inequality in coverages and opportunity gaps of nutrition interventions and their respective service delivery platforms in India, 2006 and 2016, by wealth quintile and age group

Period	Age group	Indicators	2006						2016					
			Q1	Q2	Q3	Q4	Q5	SII	Q1	Q2	Q3	Q4	Q5	SII
**During pregnancy**	**15-19y**	Early ANC	32.1	46.2	62.4	84.2	69.4	49.3***	44.1	59.4	64.0	68.9	75.0	33.5***
		IFA 100+	7.6	12.8	19.2	32.4	24.7	20.1***	16.7	28.1	31.6	35.7	42.4	26.4***
		**Gaps early ANC: IFA 100+**	24.2	33.6	43.2	51.3	43.5	28.8***	27.3	31.3	32.4	33.7	32.9	20.7***
	**20-24y**	Early ANC	39.0	59.3	71.1	80.1	83.2	58.6***	47.3	61.1	68.5	71.2	78.2	34.2***
		IFA 100+	9.4	18.7	27.5	33.8	47.1	40.5***	18.3	29.9	33.9	39.1	45.3	29.8***
		**Gaps early ANC: IFA 100+**	29.5	40.1	43.0	45.8	35.5	20.0***	28.9	31.1	34.5	31.8	32.8	7.9***
	**25-49y**	Early ANC	49.6	66.6	77.4	85.0	92.0	52.4***	48.8	62.9	69.7	75.7	80.9	33.6***
		IFA 100+	15.4	30.6	40.1	48.0	50.1	43.8***	21.7	36.6	42.1	47.5	53.5	33.3***
		**Gaps early ANC: IFA 100+**	33.9	35.2	37.1	36.8	41.8	4.8	26.9	26.2	27.5	27.9	27.3	-2.9
	**15-19y**	ANC4+	20.8	42.3	48.5	77.3	65.3	53.0***	37.5	60.9	63.5	66.1	67.1	37.3***
		Deworming	3.2	3.8	6.5	9.0	1.9	4.1	13.6	18.5	17.7	18.9	26.0	8.8**
		**Gaps ANC 4+: Deworming**	17.6	38.3	42.1	67.8	63.5	53.7***	23.8	42.3	45.7	47.7	40.7	27.4***
	**20-24y**	ANC4+	28.3	53.4	68.5	79.2	85.2	70.3***	34.0	54.1	64.6	68.5	72.5	44.1***
		Deworming	2.5	3.7	5.7	5.6	8.6	6.5***	13.1	19.4	20.7	21.1	21.3	9.3***
		**Gaps ANC 4+: Deworming**	25.7	49.4	62.4	73.4	76.4	64.3***	20.8	34.7	43.8	47.5	51.1	33.3***
	**25-49y**	ANC4+	41.6	65.0	78.4	89.1	88.9	58.5***	38.5	58.9	67.4	74.3	78.6	39.4***
		Deworming	3.5	6.4	8.1	6.8	5.2	1.7	15.6	24.0	23.9	24.9	23.0	4.4**
		**Gaps ANC 4+: Deworming**	36.9	58.3	70.1	81.9	83.6	55.1***	22.9	34.9	43.5	49.5	55.5	30.7***
	**15-19y**	THR	20.8	24.4	15.9	14.8	1.1	-5.1	56.0	65.8	61.1	56.1	38.9	-4.4
		Nutrition counseling	8.3	12.9	6.5	11.0	0.0	2.3	38.3	50.6	46.4	45.3	31.6	4.6
		**Gaps THR: Nutrition counseling**	12.5	11.5	9.5	3.8	1.1	-9.1*	17.7	15.2	14.6	10.9	7.3	-10.2***
	**20-24y**	THR	25.8	24.2	17.2	9.9	5.8	-24.1***	57.2	63.5	60.8	53.1	41.4	-17.2***
		Nutrition counseling	14.6	12.4	10.3	5.9	2.8	-13.8***	39.6	48.6	48.9	42.7	33.1	-6.1***
		**Gaps THR: Nutrition counseling**	11.2	11.8	6.9	4.1	3.0	-13.3***	17.7	15.0	11.9	10.5	8.3	-10.7***
	**25-49y**	THR	24.6	21.3	13.5	6.7	3.1	-30.1***	58.4	63.2	53.7	41.5	31.2	-40.7***
		Nutrition counseling	13.9	14.3	7.7	4.1	1.8	-18.7***	40.4	48.9	43.0	34.0	24.9	-27.9***
		**Gaps THR: Nutrition counseling**	10.6	7.0	5.8	2.6	1.3	-14.5***	18.0	14.3	10.7	7.5	6.3	-14.6***
**During delivery**	**15-19y**	Institutional delivery	29.6	50.3	72.4	86.9	87.7	63.0***	78.5	86.5	89.7	95.2	98.6	22.5***
		EIBF	16.2	27.7	23.1	30.8	34.2	20.0***	42.2	45.4	42.6	41.9	44.9	0.5
		**Gaps Institutional delivery: EIBF**	12.2	25.4	47.7	54.0	52.8	36.2***	36.6	42.1	47.5	52.5	53.8	8.1*
	**20-24y**	Institutional delivery	37.7	61.0	75.6	85.6	91.6	68.0***	77.8	88.0	92.4	95.1	97.0	22.7***
		EIBF	24.6	27.8	31.4	28.0	25.7	5.4	39.8	43.0	44.6	43.3	41.7	2.8

		**Gaps Institutional delivery: EIBF**	13.8	33.9	43.7	58.0	67.4	39.0***	39.3	45.9	48.6	52.3	55.9	4.6*
	**25-49y**	Institutional delivery	51.1	73.6	83.4	92.7	96.5	57.6***	78.4	90.6	94.9	97.3	98.9	21.8***
		EIBF	27.2	29.5	33.4	37.1	30.6	6.6	39.1	47.0	44.2	42.6	42.8	-0.2
		**Gaps Institutional delivery: EIBF**	26.9	44.7	51.6	54.9	66.6	29.5***	41.7	44.0	52.0	55.0	56.5	5.9*
**During lactation**	**15-19y**	THR	15.6	15.8	11.2	6.8	3.9	-7.7	50.8	55.5	53.6	45.7	32.5	-8.2*
		Nutrition counseling	5.7	8.3	7.3	4.2	2.5	2.2	33.2	41.1	38.5	36.9	25.1	1.1
		**Gaps THR: Nutrition counseling**	9.9	7.4	3.9	2.5	1.3	-8.9*	17.5	14.3	15.1	8.9	7.4	-7.9**
	**20-24y**	THR	20.3	17.3	12.3	7.3	3.9	-20.6***	52.5	57.4	55.0	47.8	37.2	-16.5***
		Nutrition counseling	10.4	8.3	6.2	4.4	2.6	-10.1***	34.4	42.9	43.3	38.7	29.9	-3.7*
		**Gaps THR: Nutrition counseling**	9.9	9.0	6.1	2.9	1.3	-12.2***	18.1	14.4	11.7	9.2	7.3	-12.4***
	**25-49y**	THR	22.6	17.6	11.5	3.0	2.5	-30.3***	54.0	58.6	48.2	39.1	27.9	-38.5***
		Nutrition counseling	11.6	10.8	4.9	2.6	0.9	-16.8***	36.8	44.9	38.0	32.4	22.4	-25.5***
		**Gaps THR: Nutrition counseling**	11.0	6.7	6.6	0.5	1.6	-14.4***	17.2	13.8	10.2	6.7	5.5	-12.7***
**During childhood**	**15-19y**	Weight measurement	13.2	16.5	16.1	12.8	6.4	3.4	43.4	52.8	46.9	43.7	39.6	-1.0
		Weight counseling	6.7	6.6	9.0	9.4	6.4	2.3	25.7	34.6	27.0	30.2	23.7	0.9
		**Gaps Weight: Weight counseling**	6.5	9.9	7.1	3.4	0.0	1.2	17.7	18.3	19.9	13.5	15.9	-1.9
	**20-24y**	Weight measurement	21.2	20.2	16.4	7.8	6.4	-18.3***	45.5	54.1	52.6	47.8	39.7	-6.4***
		Weight counseling	12.0	10.4	7.9	4.1	2.6	-12.1***	28.3	35.8	35.9	31.2	26.1	-2.6
		**Gaps Weight: Weight counseling**	9.2	9.8	8.5	3.8	3.8	-6.5***	17.2	18.3	16.7	16.6	13.7	-3.8***
	**25-49y**	Weight measurement	19.2	24.6	13.8	6.0	4.3	-24.8***	48.2	53.3	48.3	41.2	29.7	-29.6***
		Weight counseling	11.0	11.9	7.8	3.2	2.3	-13.9***	28.4	35.5	32.7	27.7	18.9	-18.8***
		**Gaps Weight: Weight counseling**	8.2	12.7	6.0	2.8	2.0	-11.4***	19.8	17.8	15.6	13.5	10.8	-11.0***
	**15-19y**	ORS	13.8	23.4	27.0	25.6	58.7	25.8*	37.4	48.4	37.5	36.9	60.1	6.9
		Zinc	0.0	0.0	0.0	0.0	0.0	0	18.4	16.8	12.8	23.9	38.0	7.2
		**Gaps ORS: Zinc**	13.8	23.4	26.9	25.6	58.7	25.8*	18.3	31.0	25.1	12.6	22.1	-2.5
	**20-24y**	ORS	23.5	22.7	32.7	36.3	62.8	31.6***	43.4	49.1	57.6	55.0	61.3	20.8***
		Zinc	0.2	0.6	0.0	0.0	0.0	-0.2	17.5	19.3	21.7	20.1	22.4	5.3
		**Gaps ORS: Zinc**	23.3	22.1	32.2	36.3	62.8	31.1***	25.1	29.9	35.6	35.0	38.2	10.3**
	**25-49y**	ORS	24.7	42.2	43.5	64.5	57.7	48.7***	49.5	54.4	56.5	59.0	65.8	19.1***
		Zinc	0.0	0.0	0.0	7.2	0.0	5.3	21.0	20.9	22.0	26.6	30.6	13.7**
		**Gaps ORS: Zinc**	24.7	42.2	45.2	57.3	57.7	46.2**	26.3	33.1	33.0	32.3	35.0	3.2

ANC: antenatal care; IFA: iron folic acid; EIBF: Early initiation of breastfeeding; ORS: Oral Rehydration Salts Solutions; THR: Take home ration

Supplemental Table 2: Inequality in coverages and opportunity gaps of nutrition interventions and their respective service delivery platforms in India, 2006 and 2016, by education

Period	Indicators	2006					2016				
		No education	Primary schooling	Secondary schooling	≥ High school	SII	No education	Primary schooling	Secondary schooling	≥ High school	SII
During pregnancy	Early ANC	25.5	42.4	63.3	88.4	66.7***	41.2	53.8	65.7	76.4	43.7***
	IFA 100+	5.9	13.3	24.8	49.7	41.9***	15.8	23.9	36.2	51.0	42.7***
	**Gaps early ANC: IFA 100+**	19.3	29.0	37.9	38.7	**36.6*****	25.4	30.0	29.4	25.2	**11.5*****
	ANC4+	16.0	35.5	59.2	85.3	73.2***	27.9	45.3	60.9	72.8	56.0***
	Deworming	2.4	3.5	5.4	6.5	5.5***	12.1	15.7	21.1	23.7	16.2***
	**Gaps ANC 4+: Deworming**	13.5	31.9	53.5	78.7	**68.0*****	15.7	29.5	39.7	49.0	**40.7*****
	THR	17.8	23.5	19.9	6.9	-1.1	50.2	59.0	56.7	34.8	-8.3
	Nutrition counseling	8.1	13.6	12.1	4.7	4.1***	33.0	43.7	44.7	27.8	3.8***
	**Gaps THR: Nutrition counseling**	9.7	9.9	7.9	2.2	-5.5***	17.3	15.3	12.0	7.0	-12.8***
During delivery	Institutional delivery	19.5	39.0	64.9	93.1	75.8***	63.7	75.6	89.3	97.0	44.3***
	EIBF	16.1	24.1	32.5	35.2	28.5***	38.4	42.4	46.9	42.2	9.4***
	**Gaps Institutional delivery: EIBF**	2.8	15.2	31.3	56.4	44.9***	27.5	34.8	43.0	54.9	4.2***
During lactation	THR	14.2	18.6	15.2	5.2	-1.9	45.4	53.5	51.8	31.3	-7.1***
	Nutrition counseling	6.0	9.5	8.6	3.4	2.6**	29.1	38.4	40.3	24.7	4.3***
	**Gaps THR: Nutrition counseling**	8.2	9.1	6.6	1.9	-4.7***	16.3	15.1	11.5	6.6	-11.1***
During childhood	Weight measurement	13.7	21.5	18.0	5.2	2.5*	39.0	49.9	49.5	32.1	0.9
	Weight counseling	6.4	10.8	9.5	3.3	3.1***	23.1	31.8	32.7	20.9	4.4***
	**Gaps Weight: Weight counseling**	7.3	10.6	8.4	1.9	-0.6	15.9	18.0	16.7	11.2	-3.5***
	ORS	18.6	24.5	34.4	53.1	32.4***	43.4	47.8	53.8	60.6	20.7***
	Zinc	0.1	0.5	0.3	1.6	1.0	17.3	20.6	21.9	26.3	9.8***
	**Gaps ORS: Zinc**	18.6	24.0	34.0	51.4	31.9***	25.7	27.1	31.3	34.0	13.0***

ANC: antenatal care; IFA: iron folic acid; EIBF: Early initiation of breastfeeding; ORS: Oral Rehydration Salts Solutions; THR: Take home ration

Supplemental Table 3: Inequality in coverages and c opportunity gaps of nutrition interventions and their respective service delivery platforms in India, 2006 and 2016, by wealth quintile and residential area

Period	Residence	Indicators	2006						2016					
			Q1	Q2	Q3	Q4	Q5	SII	Q1	Q2	Q3	Q4	Q5	SII
**During pregnancy**	**Rural**	Early ANC	24.8	40.2	51.9	66.9	74.7	52.2***	35.7	50.5	58.9	67.4	74.3	44.7***
		IFA 100+	6.8	11.8	18.0	29.9	40.9	27.5***	13.2	21.5	29.6	35.9	43.9	35.4***
		**Gaps early ANC: IFA 100+**	17.9	28.1	33.6	36.4	33.6	**31.6*****	22.4	29.0	29.3	31.4	30.4	**18.1*****
	**Urban**	Early ANC	33.6	47.5	55.3	70.5	83.6	54.7***	39.9	52.5	59.9	69.6	76.8	30.8***
		IFA 100+	8.1	13.0	18.2	27.2	41.6	41.4***	18.5	25.8	33.7	40.2	49.3	30.2***
		**Gaps early ANC: IFA 100+**	25.3	34.2	36.5	43.0	41.5	**16.3*****	21.4	26.9	26.3	29.4	27.4	**1.4**
	**Rural**	ANC4+	14.7	30.7	45.0	59.3	73.2	56.0***	20.7	39.5	53.0	62.2	67.5	55.6***
		Deworming	2.7	4.2	4.2	5.9	5.5	3.7***	11.4	15.8	18.9	20.2	21.5	12.6***
		**Gaps ANC 4+: Deworming**	11.9	26.4	40.4	53.1	67.3	**51.8*****	9.3	23.7	34.1	42.0	46.0	**40.8*****
	**Urban**	ANC4+	30.5	42.4	55.3	70.0	83.6	58.7***	31.6	47.1	57.7	67.1	74.1	32.6***
		Deworming	1.7	3.7	3.8	5.1	5.7	4.2***	13.6	18.4	21.0	22.0	22.8	5.1**
		**Gaps ANC 4+: Deworming**	28.9	38.7	51.3	64.7	77.6	**53.6*****	17.8	28.6	36.8	45.0	51.3	**26.0*****
	**Rural**	THR	22.1	24.2	22.2	17.6	12.0	-3.8*	52.3	61.5	65.7	61.5	53.4	6.7***
		Nutrition counseling	10.6	14.2	12.8	10.2	6.4	1.6	32.4	44.6	50.4	48.6	41.2	16.2***
		**Gaps THR: Nutrition counseling**	11.5	10.0	9.5	7.4	5.7	**-5.3*****	19.9	16.9	15.3	12.9	12.2	**-9.0*****
	**Urban**	THR	17.5	17.4	11.5	8.3	2.4	-20.2***	48.9	49.9	48.0	40.3	27.4	-32.0***
		Nutrition counseling	11.4	9.7	7.0	4.6	1.3	-12.8***	34.2	38.6	39.8	33.2	22.9	-23.9***
		**Gaps THR: Nutrition counseling**	6.1	7.7	4.5	3.7	1.1	-9.5***	14.8	11.4	8.2	7.1	4.5	-10.0***
**During delivery**	**Rural**	Institutional delivery	17.4	32.9	49.0	64.3	82.6	58.8***	60.8	74.3	82.2	89.1	94.7	40.9***
		EIBF	16.7	24.9	30.0	30.9	34.1	22.2***	39.0	42.7	44.9	45.2	44.6	7.9***
		**Gaps Institutional delivery: EIBF**	0.7	7.8	19.5	36.0	48.5	36.3***	24.8	34.0	39.5	45.5	50.8	6.0***
	**Urban**	Institutional delivery	35.5	49.5	64.9	79.5	90.5	59.6***	64.3	75.8	84.0	91.5	96.1	24.9***
		EIBF	23.6	26.9	28.4	31.5	30.5	7.5*	43.7	47.6	44.8	47.3	42.9	-4.9*
		**Gaps Institutional delivery: EIBF**	12.6	23.9	38.3	48.3	60.4	38.1***	24.5	30.3	41.2	44.6	53.8	10.2***
**During lactation**	**Rural**	THR	18.1	18.5	17.1	12.6	8.1	-5.8***	47.5	56.4	59.0	56.2	48.0	5.5***
		Nutrition counseling	8.0	10.0	9.2	7.1	4.8	0.5	28.3	39.8	44.5	43.9	36.7	15.3***
		**Gaps THR: Nutrition counseling**	10.1	8.4	7.9	5.5	3.3	**-6.2*****	19.2	16.6	14.5	12.3	11.2	**-9.2*****
	**Urban**	THR	13.8	14.0	8.5	5.8	1.7	-16.6***	48.1	46.5	44.4	36.9	24.3	-31.8***
		Nutrition counseling	8.2	6.1	4.9	3.0	0.8	-8.9***	32.3	35.7	36.2	30.3	20.6	-22.7***
		**Gaps THR: Nutrition counseling**	5.6	7.8	3.6	2.8	0.9	-8.5***	15.8	10.8	8.2	6.6	3.7	-8.9***
**During childhood**	**Rural**	Weight measurement	17.9	20.8	20.1	16.7	11.4	2.9	39.2	50.2	55.1	54.0	48.4	15.3***
		Weight counseling	8.8	10.4	9.9	8.1	6.5	0.4	22.5	31.8	36.2	35.9	30.7	13.8***

		**Gaps Weight: Weight counseling**	9.1	10.4	10.1	8.6	4.8	-0.2	16.7	18.3	18.9	18.1	17.6	1.4*
	**Urban**	Weight measurement	10.7	12.7	11.4	6.7	3.0	-12.7***	41.3	41.9	44.1	37.8	26.3	-25.6***
		Weight counseling	6.5	7.4	6.0	3.4	1.2	-8.5***	24.7	27.4	29.7	25.2	17.5	-16.7***
		**Gaps Weight: Weight counseling**	4.2	5.3	5.4	3.3	1.9	-4.4***	16.6	14.6	14.4	12.6	8.9	-9.0***
	**Rural**	ORS	18.6	22.0	33.9	44.8	45.1	28.3***	41.7	47.1	48.8	53.4	56.4	16.7***
		Zinc	0.1	0.2	0.0	1.0	1.0	0.7	17.7	18.4	21.0	21.3	22.5	6.0***
		**Gaps ORS: Zinc**	18.5	21.7	33.9	43.7	43.9	27.4***	23.6	28.0	27.1	31.7	33.9	12.3***
	**Urban**	ORS	22.2	26.0	28.7	34.8	46.4	28.9***	54.0	49.5	50.7	60.5	62.2	16.0***
		Zinc	1.6	0.7	0.7	0.1	0.3	-1.5	12.9	22.8	22.0	23.6	27.0	9.5*
		**Gaps ORS: Zinc**	20.5	25.4	28.1	34.7	46.2	28.3***	41.5	25.5	28.8	36.3	35.0	4.0

ANC: antenatal care; IFA: iron folic acid; EIBF: Early initiation of breastfeeding; ORS: Oral Rehydration Salts Solutions; THR: Take home ration

Supplemental Figure 1: Opportunity gaps during pregnancy in different states in India in 2016

a) Early ANC: IFA 100+

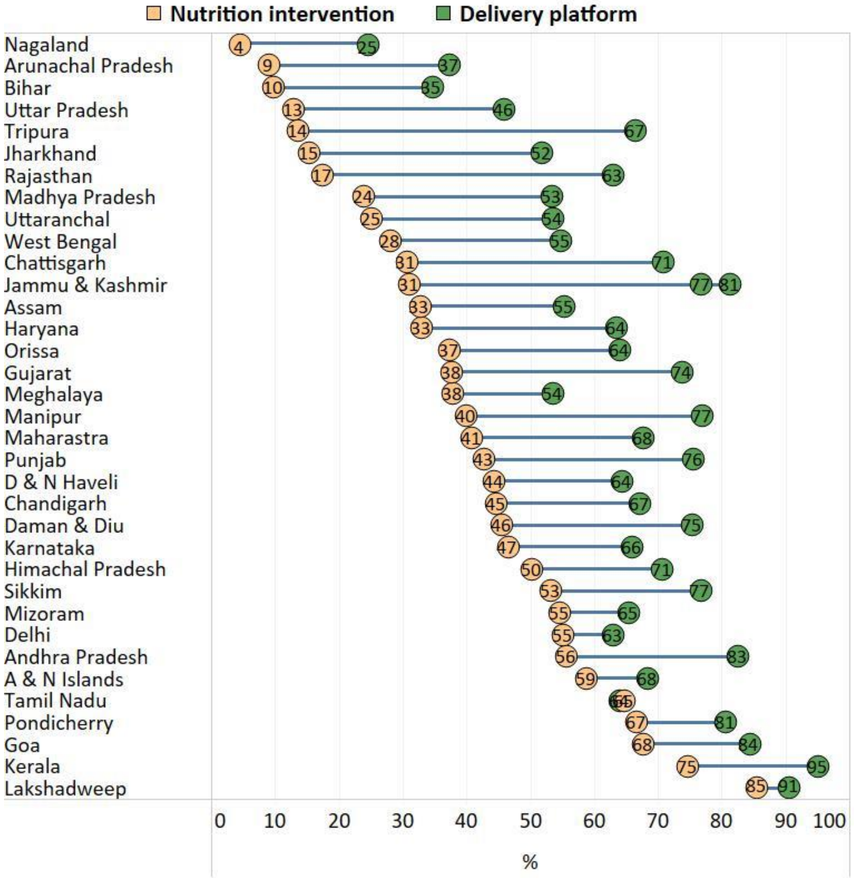

b) ANC 4+: Deworming

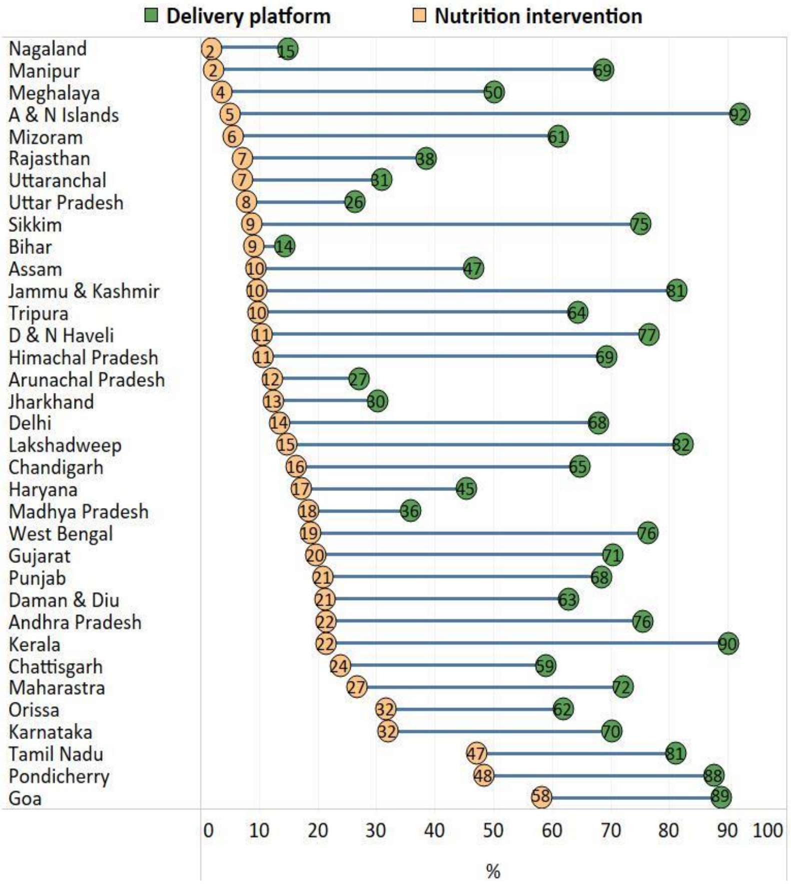

ANC: antenatal care; IFA: iron folic acid.

Supplemental Figure 2: Opportunity gaps during delivery and lactation in different states in India in 2016

a) Institutional delivery: EIBF

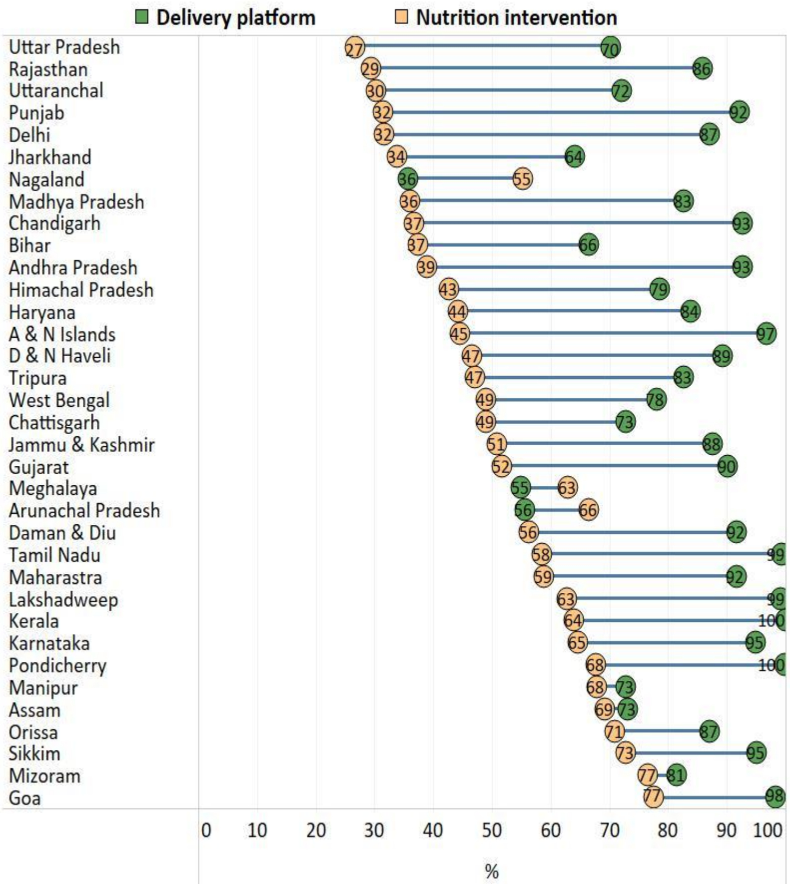

b) Food supplementation during lactation: Nutrition counseling

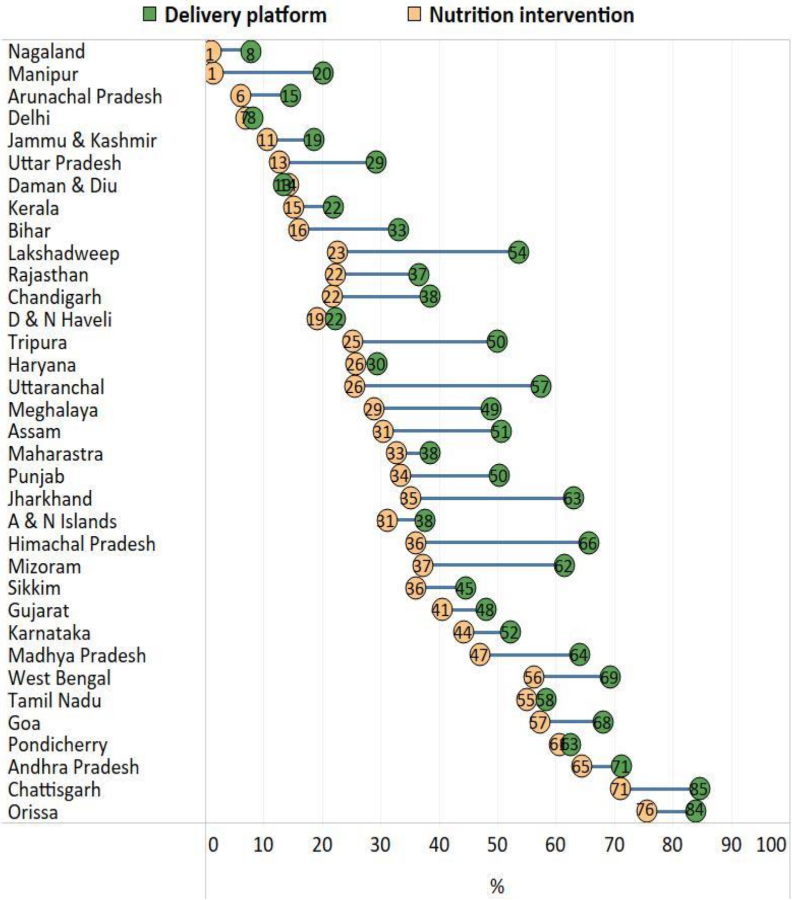

EIBF: Early initiation of breastfeeding

Supplemental Figure 3: Opportunity gaps during childhood in different states in India in 2016

a) Child weight: Weight counseling **b) ORS: Zinc**

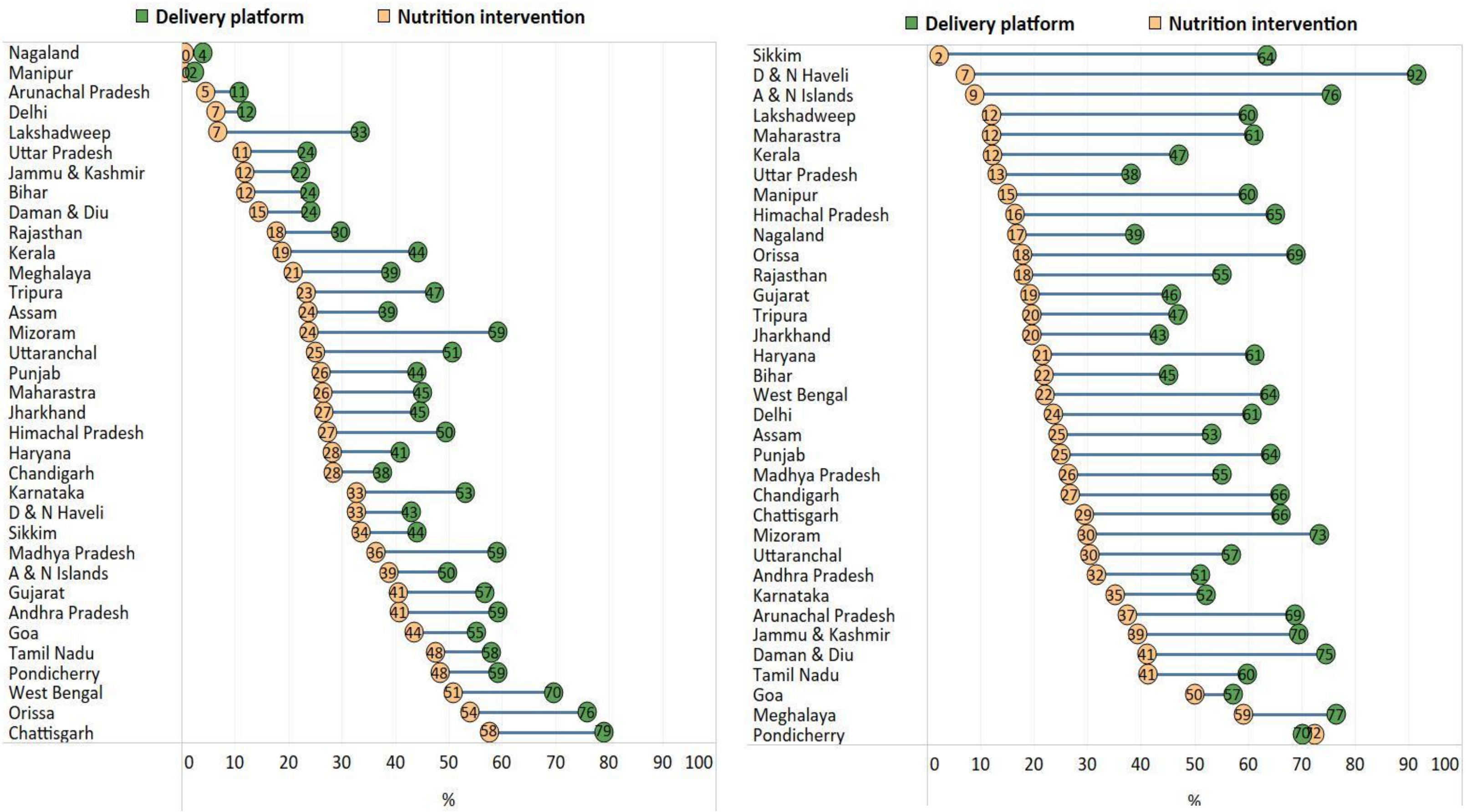

ORS: Oral Rehydration Salts Solutions.
